# Treatment of scleromyxedema with IVIg

**DOI:** 10.1186/1546-0096-10-S1-A97

**Published:** 2012-07-13

**Authors:** David W Moser, Thomas A Griffin

**Affiliations:** 1 Childrens Hospital Medical Center, Cincinnati, OH, USA; 2 Cincinnati Children's Hospital Medical Center, Cincinnati, OH, USA

## Purpose

Report findings in a case of Scerlomyxedema.

## Methods

Case report.

## Results

An 18 year old male presented with a rash of three weeks duration. Soon after the rash appeared, he developed pain and swelling of the right hand, as well as numbness and tingling of the right forearm. A recreational weight lifter, he noticed progressively decreasing stamina and amount of weight that he could lift. Swelling of the eyelids and bridge of nose prompted treatment for an allergic reaction, but these signs did not improve with antihistamines and oral corticosteroids. Family history was significant for the patient’s father and paternal grandfather both having a history of Idiopathic Thrombocytopenic Purpura. Physical exam revealed 2-3 mm skin-colored papules scattered on arms and thorax, with prominent areas at the base of neck and shoulders.

**Figure 1:**
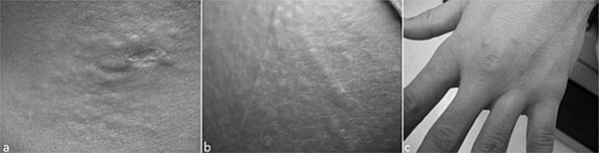


Periorbital and glabellar swelling were visible, and bogginess of the left 3rd metacarpalphalengeal joint was present. Strength was decreased in arms and legs, 3/5 and 4/5 respectively. Sensation for pin-prick and vibration was decreased in a stocking/glove distribution of the distal extremities. He had a fine tremor of the tongue and right arm. A shaved skin biopsy showed a papule with prominent mucin deposition (alcian blue staining) and increased fibroblast cellularity.

**Figure 2:**
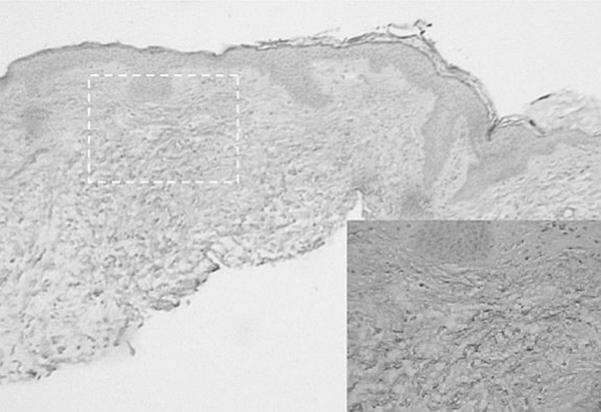


Bone marrow aspirate showed mild hypocellularity with 2-3% plasma cells (polyclonal). Serum IgE level was 884 IU/mL, however serum protein electrophoresis and immunoelectrophoresis were normal. Electromyography (EMG) was consistent with a sensory/motor peripheral polyneuropathy with demyelinating and axonal features involving the arms. Additional laboratory workup for underlying autoimmune, thyroid, neurologic, infectious and neoplastic disease was negative. Based on histopathology and clinical features, he was diagnosed with Scleromyxedema, and therapy with intravenous immunoglobulin (IVIg) was initiated with moderate symptomatic improvement after the first infusion. After three infusions, there was complete resolution of symptoms, and he had a normal physical exam without rash or neurologic abnormalities. He was treated with IVIg for a total of 6 months, and has not experienced symptoms suggesting relapse during the 6 months after discontinuation of IVIg. Repeat EMG testing, 3 and 9 months post-presentation showed significant improvement, but persistence of very mild peripheral neuropathy.

## Conclusion

To our knowledge, this patient is the youngest male reported in the literature to have Scleromyxedema without an associated autoimmune disease, and this case supports the benefit of IVIg for the treatment of Scleromyxedema.

## Disclosure

**David W. Moser:** None; **Thomas A. Griffin:** None.

